# Effect of perioperative sodium bicarbonate administration on renal function following cardiac surgery for infective endocarditis: a randomized, placebo-controlled trial

**DOI:** 10.1186/s13054-016-1591-z

**Published:** 2017-01-05

**Authors:** Jin Sun Cho, Sarah Soh, Jae-Kwang Shim, Sanghwa Kang, Haegi Choi, Young-Lan Kwak

**Affiliations:** 1 Department of Anesthesiology and Pain Medicine, Yonsei Cardiovascular Hospital, Yonsei University College of Medicine, 50 Yonsei-ro, Seodaemun-gu, Seoul, 120-752 Republic of Korea; 2 Anesthesia and Pain Research Institute, Yonsei University College of Medicine, Seoul, Republic of Korea; 3 Department of Anesthesiology and Pain Medicine, National Health Insurance Corporation Ilsan Hospital, Gyeonggi-do, Republic of Korea

**Keywords:** Infective endocarditis, Cardiac surgery, Renal function, Sodium bicarbonate

## Abstract

**Background:**

Patients with infective endocarditis (IE) have an elevated risk of renal dysfunction because of extensive systemic inflammation and use of nephrotoxic antibiotics. In this randomized, placebo-controlled trial, we investigated whether perioperative sodium bicarbonate administration could attenuate postoperative renal dysfunction in patients with IE undergoing cardiac surgery.

**Methods:**

Seventy patients randomly received sodium chloride (*n* = 35) or sodium bicarbonate (*n* = 35). Sodium bicarbonate was administered as a 0.5 mmol/kg loading dose for 1 h commencing with anesthetic induction, followed by a 0.15 mmol/kg/h infusion for 23 h. The primary outcome was peak serum creatinine (SCr) level during the first 48 h postoperatively. The incidence of acute kidney injury, SCr level, estimated glomerular filtration rate, and major morbidity endpoints were assessed postoperatively.

**Results:**

The peak SCr during the first 48 h postoperatively (bicarbonate vs. control: 1.01 (0.74, 1.37) mg/dl vs. 0.88 (0.76, 1.27) mg/dl, *P* = 0.474) and the incidence of acute kidney injury (bicarbonate vs. control: 29% vs. 23%, *P* = 0.584) were similar in both groups. The postoperative increase in SCr above baseline was greater in the bicarbonate group than in the control group on postoperative day 2 (0.21 (0.07, 0.33) mg/dl vs. 0.06 (0.00, 0.23) mg/dl, *P* = 0.028) and postoperative day 5 (0.23 (0.08, 0.36) mg/dl vs. 0.06 (0.00, 0.23) mg/dl, *P* = 0.017).

**Conclusions:**

Perioperative sodium bicarbonate administration had no favorable impact on postoperative renal function and outcomes in patients with IE undergoing cardiac surgery. Instead, it was associated with possibly harmful renal effects, illustrated by a greater increase in SCr postoperatively, compared to control.

**Trial registration:**

ClinicalTrials.gov, NCT01920126. Registered on 31 July 2013.

**Electronic supplementary material:**

The online version of this article (doi:10.1186/s13054-016-1591-z) contains supplementary material, which is available to authorized users.

## Background

For the treatment of infective endocarditis (IE), recent evidence favors early surgical intervention to reduce the risk of stroke and death [1, 2]. Surgery with cardiopulmonary bypass (CPB), however, is inevitably accompanied by numerous factors that contribute to acute kidney injury (AKI) [3]. An early, exaggerated inflammatory response with oxidative stress and the preoperative use of nephrotoxic antibiotics further predispose patients with IE to renal impairment [4–6]. Indeed, AKI affects up to 60% of patients with IE undergoing cardiac surgery [6], and the occurrence and severity of AKI have been indisputably associated with mortality and poor outcomes [7].

Sodium bicarbonate has been demonstrated to protect the kidneys through urinary alkalization and increasing the tubular pH [8, 9]. It reduces the pH-dependent conversion of hemoglobin to methemoglobin and production of free radicals via the Haber-Weiss reaction [10, 11]. After a pilot study reported that with sodium bicarbonate infusion there was a reduced incidence of AKI after cardiac surgery [8], numerous clinical trials evaluated its renoprotective efficacy in different types of surgery and patients, yielding conflicting results [12–15]. Considering the massive oxidative stress associated with systemic inflammation and prolonged use of nephrotoxic antibiotics in patients with IE [16], the abilities of sodium bicarbonate to attenuate oxidative stress may prove especially helpful in preventing AKI in these patients. However, most previous studies investigating the renal effects of sodium bicarbonate have excluded patients with systemic infection, and no study has heretofore been conducted solely in patients with IE. The aim of this prospective, randomized, placebo-controlled trial was to investigate the effect of perioperative sodium bicarbonate administration on postoperative renal function in patients with IE undergoing cardiac surgery with CPB.

## Methods

The study protocol was approved by the Institutional Review Board and Hospital Research Ethics Committee of Severance Hospital at Yonsei University College of Medicine (#4-2013-0376) and registered at ClinicalTrials.gov on July 2013 (NCT01920126). The trial was conducted at Yonsei University College of Medicine in Seoul, Korea, between August 2013 and July 2016. After providing written informed consent, 70 patients between 20 and 80 years old, who were scheduled for valvular heart surgery for IE, were enrolled. IE was diagnosed according to the modified Duke criteria [17]. Patients were excluded if they met at least one of these criteria preoperatively: (1) estimated glomerular filtration rate (eGFR) <15 ml/min/1.73 m^2^; (2) receiving renal replacement therapy; (3) receiving intra-aortic balloon pump support; (4) preexisting hypernatremia, alkalosis, or severe pulmonary edema; or (5) chronic moderate dose to high dose corticosteroid therapy (>10 mg/day prednisolone or equivalent).

Enrolled patients were randomly assigned to either the control or bicarbonate group at a 1:1 ratio using a computer-generated random-number table. Assignments were concealed in sealed envelopes. The study drugs were prepared in identical 50-ml syringes by an anesthesia nurse not involved with the study. The bicarbonate group received 154 mEq/l sodium bicarbonate with a 0.5 mmol/kg intravenous (IV) loading dose over 1 h immediately after anesthetic induction, followed by an IV continuous infusion at 0.15 mmol/kg/h over 23 h. The control group received equivalent volumes of IV 0.9% sodium chloride for the same duration. Attending surgeons and anesthesiologists involved in patient management in the operating room and intensive care unit (ICU) were blinded to the group assignment. Group assignment was disclosed after hospital discharge.

All patients received institutionally standardized anesthetic care and CPB management as previously described [18]. Intraoperatively, all patients received balanced crystalloid (Plasma solution A 1000 inj, CJ, Seoul, Korea) at 6 ml/kg/h and balanced synthetic colloid (Volulyte; Fresenius Kabi, Bad Homburg, Germany) to replace estimated blood loss (to a maximum of 500 ml). Mean arterial pressure (MAP) <70 mm Hg was treated with norepinephrine, with the infusion starting at 0.01 μg/kg/min and titrated to a maximum of 0.3 μg/kg/min. If the blood pressure was not maintained with norepinephrine, vasopressin was infused at 2.4–4.0 units/h. Milrinone (0.5 μg/kg/min) was infused when the mixed venous oxygen saturation decreased below 60% for >10 minutes. Postoperatively, when urine output was <1 ml/kg/h for 30 minutes, loop diuretics were administered after a 200-ml IV bolus of crystalloid. If this was inadequate, fluid boluses were repeated up to three times. If urine output remained inadequate 15 minutes after the third bolus, 5 mg of IV furosemide was administered and then repeated every 30 minutes, with escalating doses until the desired effect (urine output >1 ml/kg/h) was achieved. Renal replacement therapy was initiated if any of the following occurred: refractory oliguria (urine output <0.5 ml/kg/h for 12 h), fluid overload, hyperkalemia (>6.5 mEq/l), rapidly rising serum potassium level, signs of uremia, or metabolic acidosis (pH <7.10).

### Outcome measures

The primary outcome measure was the difference in peak serum creatinine (SCr) level between groups during the first 48 h postoperatively. We assumed that >0.3 mg/dl difference in SCr would be clinically significant, based on the Acute Kidney Injury Network (AKIN) criteria [19]. Secondary outcomes included the incidence of postoperative AKI (diagnosed by the AKIN criteria) [19], electrolyte abnormalities, major morbidity endpoints, ICU and hospital length of stay, and in-hospital mortality. Changes in SCr and eGFR, which were measured before surgery, upon ICU arrival, and at least once per day until postoperative day (POD) 5, were assessed. Serum sodium (Na^+^), potassium (K^+^), bicarbonate, and pH were measured before surgery and at 6 and 24 h after commencing the study drug infusion. The incidence of hypernatremia (serum (Na^+^) >150 mmol/l), hypokalemia (serum (K^+^) <3.5 mmol/l), or alkalemia (serum pH >7.50) was determined. The major morbidity endpoints were permanent stroke, hemostatic re-exploration, AKI, prolonged ventilator care (>48 h), and deep sternal wound infection.

Preoperative variables included demographics, preoperative morbidities, left ventricular ejection fraction, Cleveland Clinic score [20], and EuroSCORE. Factors associated with an increased risk of renal injury in patients with IE were investigated [6]. Intraoperative variables included the surgery duration, CPB and aortic cross-clamp times, fluid balance, transfusion requirements, and vasoconstrictor/inotropic medication requirements. Hemodynamic variables, including heart rate (HR), MAP, mean pulmonary arterial pressure (MPAP), central venous pressure (CVP), and cardiac index (CI), were recorded 15 minutes after anesthetic induction, 15 minutes after weaning from CPB, at sternum closure, upon ICU arrival, and at 24 h and 48 h after surgery. Postoperative variables included fluid balance, urine output, blood loss (chest tube drainage), packed erythrocyte transfusion, and vasoconstrictor/inotrope usage during the first 48 h after surgery. Body temperature, C-reactive protein level, white blood cell count, and percentage of neutrophils were measured before surgery and at least once per day until POD 5.

### Statistical analysis

The standard deviation (SD) of SCr level at 48 h postoperatively was previously reported as 0.4 in patients undergoing valvular heart surgery at our institute [18]. We estimated that 29 patients in each group would be required to detect a mean difference of 0.3 and SD of 0.4 in peak SCr levels during the first 48 h postoperatively, with 80% power at a significance level of *P* <0.05. Factoring in a 20% dropout rate, we enrolled 35 patients in each group.

Data are shown as mean ± SD, median (interquartile range), or number. Between-group comparisons of continuous variables were performed using the independent *t* test or Mann-Whitney *U* test, after testing for normality using the Kolmogorov-Smirnov test. Dichotomous variables were compared using chi-square test or Fisher’s exact test. Serially measured variables were analyzed using a linear mixed model with patient indicator as a random effect and group, time, and group-by-time as fixed effects. This was followed by post hoc analysis using the Bonferroni correction. All statistical analyses were two-tailed and performed using SPSS 20 software (SPSSFW, SPSS Inc., IBM, Armonk, NY, USA). *P* <0.05 was considered statistically significant.

## Results

Of the 70 patients enrolled, IE was definitively confirmed through surgical findings and pathologic examination in 32 patients in each group. According to the intention-to-treat principle, statistical analyses were performed using data from all enrolled patients. Four patients in each group (*P* > 0.999) underwent emergency surgery within 24 h after being diagnosed with IE. All patients were treated with antibiotics before the day of surgery. At the time of surgery, 29 (83%) and 27 (77%) patients in the bicarbonate and control groups, respectively, had active infection with a positive blood culture, leukocytosis (>10,800/μl), fever (temperature >38 °C), or elevated C-reactive protein (>8 mg/l) (*P* = 0.550). The time between diagnosis of IE and surgery was similar in both groups (bicarbonate vs. control: 11 (5, 17) days vs. 8 (5, 17) days, *P* = 0.857). All baseline patient characteristics were comparable in the two groups, except that more patients received anti-platelet drugs prior to surgery (5 vs. 0, *P* = 0.020) and had platelet counts below 150,000/μl (11 vs. 3, *P* = 0.017) in the control group (Table 1).

**Table 1:** Demographic and perioperative clinical data

	Control group (*n* = 35)	Bicarbonate group (*n* = 35)	*P* value
Demographics			
Age (years)	55.1 ± 15.7	53.9 ± 14.1	0.738
Sex (male:female)	27:8	21:14	0.122
Body mass index (kg/m^2^)	21.6 ± 3.6	22.0 ± 3.3	0.613
Cormobidities			
Hypertension	10	10	>0.999
Diabetes mellitus	6	4	0.495
Congestive heart failure	1	0	0.314
Chronic kidney disease (SCr >1.4 mg/dl)	7	8	0.771
MI within 1 month	0	0	
Cerebrovascular disease	5	3	0.452
COPD	0	0	
Repeat surgery	4	6	0.495
Preoperative condition			
Left ventricular ejection fraction (%)	63.9 ± 8.5	65.9 ± 7.4	0.312
EuroSCORE	4 (2, 9)	5 (3, 8)	0.449
Cleveland Clinic score^a^	2 (1, 3)	2 (1, 2)	0.360
Vancomycin or aminoglycoside	27	29	0.550
Contrast use 48 h prior to surgery	19	14	0.231
Hemoglobin below 10 g/dl	14	8	0.122
Platelets below 100,000/μl /150,000/μl	3/ 11	1/ 3	0.303/0.017
Embolic events	8	13	0.192
Prosthetic valve infection	4	6	0.495
Active infection^b^/emergency^c^	27/ 4	29/ 4	0.555/>0.999
Medications			
ACEi/ARB	8	8	>0.999
β-blockers	7	8	0.771
Calcium channel blockers	3	4	0.690
Diuretics	12	14	0.621
Antiplatelet drugs	5	0	0.020
Operation			
Aortic valve replacement	11	7	0.274
Mitral valve replacement	17	19	0.632
Double valve operation	4	2	0.393
Tricuspid annuloplasty	2	3	0.643
Valve + aorta	1	4	0.164

Values are mean ± standard deviation, number of patients, or median (interquartile range). *SCr* serum creatinine, *MI* myocardial infarction, *COPD* chronic obstructive lung disease, *ACEi* angiotensin converting enzyme inhibitors, *ARB* angiotensin receptor blockers, *CABG* coronary artery bypass graft; ^a^Cleveland Clinic Foundation Acute Renal Failure Scoring System (minimum score = 0; maximum score = 17); ^b^active infection, patients exhibiting an active infection with a positive blood culture, leukocytosis (>10,800/μl), fever (>38 °C), or C-reactive protein >8 mg/l; ^c^emergency, patients undergoing emergency operation within 24 h after being diagnosed with infective endocarditis

### Renal outcomes

The peak SCr level during the first 48 h postoperatively was not significantly different between groups (bicarbonate vs. control: 1.01 (0.74, 1.37) mg/dl vs. 0.88 (0.76, 1.27) mg/dl, *P* = 0.474). The postoperative increase in SCr above baseline was significantly greater in the bicarbonate group than in the control group on POD 2 (0.21 (0.07, 0.33) mg/dl vs. 0.06 (0.00, 0.23) mg/dl, *P* = 0.028) and POD 5 (0.23 (0.08, 0.36) mg/dl vs. 0.06 (0.00, 0.23) mg/dl, *P* = 0.017). Postoperative SCr levels were higher and eGFR values were lower in the bicarbonate group than in the control group, but the group × time interactions for the SCr level and eGFR during the five PODs were not statistically significant between groups in the linear mixed-model analysis (*P* = 0.055 and 0.073, respectively).

There were no differences in the incidence of AKI (bicarbonate vs. control: 29% vs. 23%, *P* = 0.584) or distribution of AKIN stages (*P* = 0.863) between groups (Table 2). No patient, except one who received renal replacement therapy in the control group, was oliguric (<0.5 ml/kg/h) for more than 6 h and thus fulfilled the definition of AKI according to the urine output-based AKIN criteria. Of note, using another set of diagnostic criteria for AKI [8, 15, 21] (increase in SCr >25% or 0.5 mg/dl from baseline), the incidence of AKI was significantly higher in the bicarbonate group than in the control group (60% vs. 31%, *P* = 0.016).

**Table 2:** Incidence of postoperative acute kidney injury, serum creatinine level and glomerular filtration rate

	Control group (*n* = 35)	Bicarbonate group (*n* = 35)	*P* value
Serum creatinine (mg/dl)^*^			
before surgery	0.90 ± 0.36	0.83 ± 0.24	0.055
immediately after surgery	0.83 ± 0.36^†^	0.73 ± 0.22^†^	
at 24 h after surgery	0.99 ± 0.35^†^	1.01 ± 0.41^†^	
at 48 h after surgery	0.94 ± 0.38	1.13 ± 0.65^†^	
at 72 h after surgery	0.89 ± 0.38	1.08 ± 0.70^†^	
at 120 h after surgery	0.84 ± 0.32	0.99 ± 0.65	
Glomerular filtration rate (ml/min per 1.73 m^2^)^*^			
before surgery	89.6 ± 34.6	94.1 ± 36.9	0.073
immediately after surgery	96.5 ± 43.4^†^	102.4 ± 42.2	
at 24 h after surgery	79.3 ± 35.1^†^	79.6 ± 39.3^†^	
at 48 h after surgery	85.7 ± 40.5	75.2 ± 38.6^†^	
at 72 h after surgery	91.1 ± 40.3	81.2 ± 40.0^†^	
at 120 h after surgery	94.7 ± 36.8	86.0 ± 42.4	
AKIN classification			
None (*n*)	27 (77%)	25 (71%)	0.863
Stage 1 (*n*)	6 (17%)	6 (17%)	
Stage 2 (*n*)	1 (3%)	2 (6%)	
Stage 3 (*n*)	1 (3%)	2 (6%)	
Any category	8 (23%)	10 (29%)	0.584

Values are mean ± standard deviation or numbers (%). Postoperative acute kidney injury (AKI) was diagnosed according to the Acute Kidney Injury Network (AKIN) classification. ^*^ *P* value demonstrates the value for the group × time interaction in the linear mixed model; ^†^ *P* < 0.05 vs. baseline value

### Fluid balance, vasopressor/inotrope requirements, electrolytes, and hemodynamics

Intraoperative fluid balance, transfusion requirements, and use of vasoconstrictor/inotropic medications were similar in both groups, except that fewer patients received platelet transfusions in the bicarbonate group (*P* = 0.023) (Table 3). Changes in perioperative hemodynamic variables, including HR (*P* = 0.699), MAP (*P* = 0.950), MPAP (*P* = 0.361), CVP (*P* = 0.409), and CI (*P* = 0.939), were comparable in the two groups in the linear mixed-model analysis (see Additional file 1 for more detail).

**Table 3:** Intraoperative data

	Control group (*n* = 35)	Bicarbonate group (*n* = 35)	*P* value
Operation time (minutes)	227.9 ± 67.0	211.8 ± 94.5	0.412
Anesthesia time (minutes)	270.7 ± 78.0	265.5 ± 105.6	0.815
Cardiopulmonary bypass time (minutes)	118.7 ± 51.8	109.6 ± 75.1	0.554
Aortic cross-clamp time (minutes)	87.3 ± 43.3	77.6 ± 56.5	0.424
Fluid balance			
Crystalloid (ml)	1000 (775, 1200)	1000 (600, 1375)	0.995
Colloid (ml)	130 (100, 300)	100 (100, 200)	0.288
Urine output (ml)	870 (560, 1240)	1000 (600, 1250)	0.526
Ultrafiltration during CPB (ml)/ *n* ^a^	2000 (1250, 2400)/25	1550 (1300, 2100)/26	0.216/0.788
Transfusion			
Amount of cell saver (ml)	500.0 (477.5, 740.0)	500.0 (480.0, 730.0)	0.652
Amount of erythrocyte (unit)	1 (0, 2)	1 (0, 2)	0.916
Patients transfused with erythrocyte	21	21	>0.999
Patients transfused with FFP	18	17	0.811
Patients transfused with PLT	12	4	0.023
Vasopressor/ Inotrope needs			
Norepinephrine dose (μg/kg/min)	0.03 (0.01, 0.04)	0.02 (0.01, 0.05)	0.317
Patients needed vasopressin	16	14	0.629
Patients needed milrinone	4	4	>0.999

Values are mean ± standard deviation, median (interquartile range), or number of patients. ^a^Number of patients receiving ultrafiltration during cardiopulmonary bypass (CPB). *FFP* fresh frozen plasma, *PLT* platelet

Postoperative fluid balance, transfusion requirements, and use of vasoconstrictor/inotropic medications during the first 48 h after surgery were comparable in both groups, except that fewer patients received erythrocyte transfusions during the first 24 h after surgery in the bicarbonate group (*P* = 0.048) (Table 4). The incidence rates of alkalemia (20% vs. 11%, *P* = 0.324), hypernatremia (0% vs. 3%, *P* = 0.314), and hypokalemia (17% vs. 14%, *P* = 0.743) during the five PODs were similar in both groups.

**Table 4:** Postoperative input, output, and vasoconstrictor use

	Control group (*n* = 35)	Bicarbonate group (*n* = 35)	*P* value
Crystalloid input (ml)			
during postoperative 24 h	2550 (2000, 3374)	2250 (1850, 3170)	0.226
during postoperative 48 h	1950 (1420, 2565)	1990 (1065, 2550)	0.450
Colloid input (ml)			
during postoperative 24 h	340 (100, 500)	280 (0, 420)	0.157
during postoperative 48 h	0 (0, 0)	0 (0, 0)	0.196
Urine output (ml)			
during postoperative 24 h	3225 (2420, 3610)	2790 (2205, 3530)	0.375
during postoperative 48 h	2800 (2190, 3515)	2490 (1700, 3915)	0.597
Chest tube drainage (ml)			
during postoperative 24 h	230 (150, 523)	200 (144, 311)	0.372
during postoperative 48 h	160 (90, 336)	130 (70, 280)	0.269
Erythrocyte (unit) transfusion			
during postoperative 24 h (*n*)^a^	0 (0, 1) (17)	0 (0, 1) (9)	0.076 (0.048)
during postoperative 48 h (*n*)^a^	0 (0, 0) (4)	0 (0, 0) (5)	0.724 (0.721)
Norepinephrine (μg/kg/min)			
during postoperative 24 h	0.02 (0.01, 0.03)	0.02 (0.01, 0.05)	0.860
during postoperative 48 h	0.02 (0.01, 0.02)	0.04 (0.04, 0.08)	0.200
Patients needed milrinone (*n*)			
during postoperative 24 h	9	6	0.382
during postoperative 48 h	3	4	0.690

Values are mean ± standard deviation, median (interquartile range), or number of patients. ^a^Number of patients requiring transfusion

### Non-renal postoperative outcomes

Changes in body temperature (*P* = 0.621), C-reactive protein level (*P* = 0.611), white blood cell count (*P* = 0.511), and percentage of neutrophils (*P* = 0.539) during the five PODs were similar in both groups (see Additional file 2 for more detail). The incidence of a composite of morbidity endpoints (*P* = 0.621) and the ICU and hospital length of stay were also similar in the two groups. Two patients in the control group died during their hospital stay because of cerebral hemorrhage, whereas no mortality occurred in the bicarbonate group (Table 5).

**Table 5:** Postoperative outcomes

	Control group (*n* = 35)	Bicarbonate group (*n* = 35)	*P* value
Composite of morbidity endpoints	14 (40%)	12 (34%)	0.621
Permanent stroke	3 (9%)	0	0.077
Hemostatic reoperation	5 (14%)	4 (11%)	0.721
Acute kidney injury	8 (23%)	10(29%)	0.584
Ventilator care >48 h	2 (6%)	2 (6%)	>0.999
Deep sternal wound infection	1 (3%)	1 (3%)	>0.999
In-hospital mortality	2 (6%)	0	0.151
Duration of ventilator care (h)	17 (13, 20)	15 (10, 22)]	0.448
Duration of intensive care unit stay (days)	2 (2, 3)	2 (1, 3)	0.361
Duration of hospital stay (days)	28 (17, 35)	26 (15, 32)	0.350

Values are number of patients (%) or median (interquartile range)

## Discussion

In this prospective, randomized, placebo-controlled trial we did not observe any beneficial effects of perioperative bicarbonate administration on postoperative renal function in patients with IE undergoing cardiac surgery with CPB. Sodium bicarbonate did not attenuate the peak postoperative SCr level nor reduce the incidence of AKI defined by the AKIN criteria. In fact, the postoperative increase in SCr above baseline was even greater in the bicarbonate group than in the control group.

Acute IE is a challenging disease that is associated with high morbidity and mortality. Although most patients with IE undergo medical treatment, more than 20% require surgery, and the number of surgical treatments has gradually increased as accumulating evidence supports the use of cardiac surgery to improve outcomes [1, 2]. However, morbidity and mortality following cardiac surgery in patients with IE remain significant. AKI is one of the most common morbidities, with a rate of 20–60% [6, 22]. Moreover, AKI clearly increases long-term postoperative mortality and cardiac event rates in patients with IE [6, 23], but definitive strategies to prevent AKI in this patient population have not been established.

The systemic inflammation and oxidative stress associated with septic conditions predispose patients with IE undergoing cardiac surgery to AKI [5]. Furthermore, prolonged use of nephrotoxic antibiotics preoperatively, and administration of contrast media shortly before cardiac surgery, adds to the risk of AKI by enhancing proinflammatory oxidative stress [16, 24]. Sodium bicarbonate administration produces blood and urine alkalization and may slow pH-dependent Haber-Weiss free-radical production, thereby attenuating reactive oxygen species-mediated tubular injury [11, 24, 25]. Sodium bicarbonate may also directly scavenge other reactive species [26]. Tubular alkalosis achieved by sodium bicarbonate has been demonstrated to mitigate the conversion of hemoglobin to methemoglobin, preventing tubular obstruction and tubular cell necrosis [10]. Accordingly, sodium bicarbonate administration has shown benefits in experimental models of ischemic [27], doxorubicin-induced [28], and contrast-medium-induced AKI [29]. However, several trials and meta-analyses investigating the renal effects of sodium bicarbonate in the context of cardiac surgery yielded inconsistent results [8, 14, 15, 21]. Based on the theoretical ability of sodium bicarbonate to reduce oxidative stress in renal tubular cells, we investigated whether perioperative sodium bicarbonate infusion would lessen renal injury in this patient population, an issue that has not been previously addressed.

Contrary to our expectation, sodium bicarbonate infusion did not demonstrate renoprotective effects in this study. The postoperative peak SCr level and incidence of AKI defined by the AKIN criteria were similar in both groups. Indeed, when AKI was diagnosed using the criteria that were applied in the first trial reporting renoprotective effects of sodium bicarbonate in cardiac surgery (increase in SCr >0.5 mg/dl or 25% above baseline) [8], the incidence of AKI was significantly greater in the bicarbonate group than in the control group. Moreover, the increase in postoperative SCr levels above baseline was significantly greater in the bicarbonate group than in the control group.

These results imply that the effects of sodium bicarbonate on renal function may not just be neutral, but they may even be harmful in patients with IE undergoing cardiac surgery, which argues against the routine use of sodium bicarbonate. Notably, intraoperative platelet transfusion requirement was higher in the control group than in the bicarbonate group, which might be attributed to the fact that more patients received anti-platelet drugs (5 vs. 0, *P* = 0.020) and had platelet counts below 150,000/μl (11 vs. 3, *P* = 0.017) in the control group. In addition, packed erythrocyte transfusion requirement during the 24 h postoperatively was also higher in the control group than in the bicarbonate group. Erythrocyte and platelet transfusions are associated with an increased risk of AKI and dialysis [30, 31]. Thus, the higher incidence of AKI in the bicarbonate group despite less transfusion further supports the possibly harmful impact of bicarbonate infusions.

In the context of cardiac surgery, previous findings demonstrate that the renoprotective effects of sodium bicarbonate were limited to low-risk patients [12, 32]. Furthermore, a recent trial of prophylactic sodium bicarbonate therapy to prevent AKI in high-risk patients was terminated early because of increased mortality in the bicarbonate group [15]. This difference according to the patient’s risk is consistent with our results, as our patients had a high risk of AKI because of infection, severe inflammation, receipt of nephrotoxic antibiotics, and surgery-related injury. The likelihood of postoperative AKI is higher in patients with more risk factors [6], and a single treatment strategy might be insufficient to attenuate renal injury in patients with multiple risk factors. This might be the most cogent explanation for our results.

The inability of sodium bicarbonate to reduce AKI was discussed from the perspective of alkalemia and resultant physiologic changes in previous studies [13–15]. Alkalemia can lead to impaired tissue perfusion [33, 34] and oxygen delivery [35], and to increased cell death and apoptosis associated with superoxide formation [36]. Most patients enrolled in our study had active inflammation and received prolonged treatment with multiple nephrotoxic antibiotics, which would aggravate renal injury associated with increased renal vascular resistance and tubular cell impairment [5, 37, 38]. During active systemic inflammation, increased renal vascular resistance (uncoupled from decreased systemic vascular resistance) may diminish renal perfusion, thereby causing ischemia [5, 38]. Indeed, multiple areas of renal infarction were observed in an animal model of IE, suggesting hypoperfusion-induced ischemia [39]. Nephrotoxic antibiotics have also been demonstrated to reduce renal blood flow by increasing renal vascular resistance rather than by lowering perfusion pressure [40]. Alkalosis following sodium bicarbonate infusion could further increase renal vascular resistance [34] and exacerbate renal tubular injury by producing intracellular acidosis. A rapid influx of CO_2_ following sodium bicarbonate infusion could worsen intracellular acidosis [41], accelerating the influx of sodium and calcium, and causing cell swelling and renal tubule dysfunction [42].

There has been a paucity of randomized, controlled trials in patients with IE undergoing cardiac surgery. This may be at least partly because these patients are often unstable or require urgent surgery, leading to a preponderance of observational studies. Thus, the primary strength of this study is that it is the first to address the renoprotective efficacy of sodium bicarbonate through randomization, solely in patients with IE undergoing cardiac surgery, although the sample size was not sufficient to compare the incidence of AKI, which is the most clinically relevant index.

The limitations of this study are as follows. First, we did not directly measure urine pH to confirm urinary alkalization by IV sodium bicarbonate administration. However, both plasma and urinary alkalization were achieved in previous studies using similar doses and durations of sodium bicarbonate infusion [8, 15, 21]. Second, we can only speculate on the reasons for the lack of beneficial effect of sodium bicarbonate in this study, because no mechanistic investigations were conducted. Third, we used balanced 130/0.4 hydroxyethyl starch solution (Volulyte) for volume replacement, upon which concerns have been raised regarding its nephrotoxicity in patients with sepsis. However, its influence on renal function is far more controversial when used in the operating theatre. Indeed, there is solid evidence favoring its use in the presence of overt volume loss [43–45]. Furthermore, the median volume used in this study did not exceed 500 ml in either group throughout the perioperative 24 h, without any intergroup difference. Thus, its influence on the observed results should be negligible.

## Conclusions

Perioperative sodium bicarbonate administration did not improve postoperative renal function or other outcomes in patients with IE undergoing cardiac surgery. Rather, it was associated with potentially harmful renal effects, as demonstrated by a greater postoperative increase in SCr above baseline, compared to control. Further research on renoprotective strategies in patients with IE is warranted.

## Additional files

Additional file 1: Perioperative hemodynamic variables. Changes in heart rate, mean blood pressure, mean pulmonary arterial pressure, and central venous pressure during the perioperative period. (PDF 48 kb)
Additional file 2: Perioperative body temperature and laboratory findings. Change in blood temperature, C-reactive protein, white blood cell count, and neutrophils during the perioperative period. (PDF 115 kb)

## Abbreviations

AKI: acute kidney injury
AKIN: Acute Kidney Injury Network
CI: cardiac index
CPB: cardiopulmonary bypass
CVP: central venous pressure
eGFR: estimated glomerular filtration rate
HR: heart rate
ICU: intensive care unit
IE: infective endocarditis
IV: intravenous
MAP: mean arterial pressure
MPAP: mean pulmonary arterial pressure
POD: postoperative day
SCr: serum creatinine
SD: standard deviation
