# Supplementary material for: Effect of perioperative sodium bicarbonate administration on renal function following cardiac surgery for infective endocarditis: a randomized, placebo-controlled trial
Source: Crit Care. 2017 Jan 5;21:3. doi: 10.1186/s13054-016-1591-z (PMC5217446; doi:10.1186/s13054-016-1591-z)
Supplement: Additional file 1: — Perioperative hemodynamic variables. Changes in heart rate, mean blood pressure, mean pulmonary arterial pressure, and central venous pressure during the perioperative period. (PDF 48 kb) [file 13054_2016_1591_MOESM1_ESM.pdf]

Table. Perioperative hemodynamic variables

Variables	Time points	Control group (n = 35)	Bicarbonate group (n = 35)	$P_{Group\ x\ Time}$
Heart rate (beat/min)	T0	72.7 ± 14.2	75.5 ± 13.7	0.699
	T1	86.2 ± 13.4*	89.1 ± 13.0*	
	T2	88.9 ± 11.3*	91.0 ± 10.6*	
	T3	87.8 ± 13.8*	92.9 ± 14.2*	
	T4	83.6 ± 15.4*	84.7 ± 15.0*	
	T5	82.0 ± 12.4*	87.2 ± 11.4*	
Mean blood pressure (mmHg)	T0	72.3 ± 9.6	74.2 ± 9.1	0.950
	T1	68.0 ± 7.8*	69.6 ± 9.3*	
	T2	72.4 ± 8.3	75.0 ± 9.3	
	T3	79.6 ± 11.4*	82.5 ± 10.8*	
	T4	80.7 ± 10.4*	83.1 ± 10.9*	
	T5	82.4 ± 7.1*	83.7 ± 11.8*	
Mean pulmonary artery pressure (mmHg)	T0	23.9 ± 10.2	22.4 ± 7.2	0.361
	T1	19.3 ± 4.6*	19.7 ± 4.5*	
	T2	20.7 ± 4.8*	20.5 ± 4.2	
	T3	18.2 ± 4.5*	19.4 ± 5.9*	
	T4	17.7 ± 4.7*	17.7 ± 4.8*	
	T5	19.0 ± 6.1*	21.3 ± 5.8	
Central venous pressure (mmHg)	T0	10.3 ± 3.6	9.6 ± 2.9	0.409
	T1	9.7 ± 2.4	10.3 ± 3.2	
	T2	11.6 ± 2.5*	11.8 ± 2.4*	
	T3	7.6 ± 2.6*	8.6 ± 3.2	
	T4	9.0 ± 3.5*	8.8 ± 3.0	
	T5	9.3 ± 3.0	10.5 ± 3.8	

Cardiac index (l/min/m^2)	T0	2.6 ± 0.6	2.6 ± 0.8	0.939
	T1	2.8 ± 0.7	2.8 ± 0.6	
	T2	2.7 ± 0.6	2.7 ± 0.7	
	T3	3.0 ± 0.6*	3.2 ± 1.0*	
	T4	3.3 ± 0.5*	3.2 ± 0.7*	
	T5	3.0 ± 0.6	3.0 ± 0.4	

Values are mean ± standard deviation.

T0, before surgery; T1, after cardiopulmonary bypass; T2, at sternum closure; T3, at intensive care unit arrival; T4, 24 h after surgery; T5, 48 h after surgery; $P_{Group \times Time}$, P-value for the group × time interaction in the linear mixed model.

* $P < 0.05$ vs. baseline value.
