# Supplementary material for: Effect of perioperative sodium bicarbonate administration on renal function following cardiac surgery for infective endocarditis: a randomized, placebo-controlled trial
Source: Crit Care. 2017 Jan 5;21:3. doi: 10.1186/s13054-016-1591-z (PMC5217446; doi:10.1186/s13054-016-1591-z)
Supplement: Additional file 2: — Perioperative body temperature and laboratory findings. Change in blood temperature, C-reactive protein, white blood cell count, and neutrophils during the perioperative period. (PDF 115 kb) [file 13054_2016_1591_MOESM2_ESM.pdf]

Table. Perioperative body temperature and laboratory findings

Variables	Time points	Control group (n = 35)	Bicarbonate group (n = 35)	$P_{Group \times Time}$
Body temperature (℃)	Before surgery	37.0 ± 0.6	37.0 ± 0.6	0.621
	POD 1	37.7 ± 0.5*	37.7 ± 0.4*	
	POD 2	37.5 ± 0.4*	37.5 ± 0.4*	
	POD 3	37.2 ± 0.5	37.5 ± 0.4*	
	POD 5	37.2 ± 0.5	37.2 ± 0.4	
C-reactive protein (mg/l)	Before surgery	45.7 ± 41.3	32.9 ± 29.3	0.611
	POD 1	88.5 ± 35.0*	88.8 ± 39.7*	
	POD 2	171.4 ± 46.4*	186.0 ± 58.6*	
	POD 3	124.0 ± 58.9*	132.7 ± 43.8*	
	POD 5	69.8 ± 54.0*	69.4 ± 35.7*	
White blood cell (10^{-3}/μl)	Before surgery	8.90 ± 3.96	7.65 ± 3.97	0.511
	POD 1	14.32 ± 5.86*	16.15 ± 7.02*	
	POD 2	13.33 ± 5.08*	14.84 ± 5.71*	
	POD 3	10.15 ± 3.21*	11.22 ± 3.74*	
	POD 5	7.47 ± 2.83*	7.99 ± 2.62	
Neutrophil (%)	Before surgery	71.4 ± 12.4	69.4 ± 12.5	0.539
	POD 1	88.5 ± 3.4*	88.7 ± 4.4*	
	POD 2	85.5 ± 4.2*	84.9 ± 5.1*	
	POD 3	80.1 ± 5.6*	80.9 ± 6.3*	
	POD 5	69.5 ± 9.5	70.5 ± 9.4	

Values are mean ± standard deviation.

POD, postoperative day; $P_{Group \times Time}$, P-value for the group × time interaction in the linear mixed model.

* $P < 0.05$ vs. baseline value
